# Recycling End-of-Life Bituminous Membranes in Asphalt Mixtures: A Laboratory Study

**DOI:** 10.3390/ma18092035

**Published:** 2025-04-29

**Authors:** Marco Pasetto, Safeer Haider, Andrea Baliello, Emiliano Pasquini

**Affiliations:** Department of Civil, Environmental and Architectural Engineering (ICEA), University of Padova, via Marzolo, 9, 35131 Padova, Italy; marco.pasetto@unipd.it (M.P.); safeer.haider@studenti.unipd.it (S.H.); emiliano.pasquini@unipd.it (E.P.)

**Keywords:** circular economy, recycling, bituminous membranes, smart infrastructures, bituminous mixtures

## Abstract

The circular economy (i.e., reuse and recycling of waste materials) is gaining attention for the goal of achieving net-zero waste. In this regard, the use of waterproofing membrane waste in bituminous materials can be a valid option, as every year, a lot of bituminous membrane wastes are generated both as production scraps or end-of-life wastes. Given this background, the recycling feasibility of end-of-life bituminous membrane waste (MW) in asphalt mixtures was assessed in this research study. To this aim, MW shreds (≤20 mm) were added to dense-graded bituminous mixtures using the dry-mixing method. The shreds were dosed at 0.5% by the mix weight (mix coded as SH−) or at 2% by mix weight (mix coded as SH+). A corresponding reference mix without MW was also tested for comparison purposes. The mixtures’ workability, strength and stiffness as well as permanent deformation, moisture and fatigue resistance were evaluated. Overall, the laboratory experimental findings showed that MW-modified bituminous mixtures with a higher dosage of membrane waste (SH+) have relatively higher moisture resistance, fatigue resistance, stiffness and high-temperature performance with respect to the corresponding reference mix. Moreover, both the reference and MW-modified mixtures showed similar workability regardless of the MW content.

## 1. Introduction

The circular economy plays a key role in reusing and recycling waste materials to achieve net-zero waste. Various types of waste materials can be used in replacement of traditional/natural pavement materials to build sustainable transport infrastructures, thus saving natural resource consumption (i.e., mineral aggregates, bitumen and fillers) [1,2,3,4,5] and limiting greenhouse gas emissions originated during landfill incineration, as well as during road constructions [6,7,8]. Hence, pavements play a significant role in the sustainability potential within the global physical assets of road infrastructures [9]. According to a waterproofing membrane market growth research report for the years 2023–2028, end-of-life roofing, walls, building structures, tunnels and bridges generate huge amounts of waterproofing membrane waste (MW) every year. However, the continuous growth rate of the waterproofing membrane market is also expected to produce a huge amount of landfill disposal [10,11].

In roofing systems, waterproofing membranes were first used in Europe in the 1960s and were also adopted in North America beginning in 1975. Waterproofing membranes generally consist of bitumen (waterproofing non-renewable agent), polymers (with elastomeric and/or plastomeric properties), glass/polyester fibers (acting as reinforcement), mineral granules (providing resistance against ultraviolet radiation), filler (to confer stiffness) and polypropylene foil (to avoid stickiness during storage of membranes) [7,12,13]. Since waterproofing bituminous membranes contain more than 50% polymer-modified bitumen, the use of MW in asphalt mixtures (which cover approximately 90% of road pavements worldwide) can promote durable and sustainable flexible pavements while endorsing the circular economy as a valuable option [8,11,14,15,16,17,18] while also considering the possible structural contribution of fibers.

MW can be generated during industrial production, installation of roofing system or at the end of service life [19]. End-of-life wastes coming from roofing demolition (named also tear-off wastes) basically differ from the production and installation ones due to the possible presence of debris (plastic, wood, nails, etc.) and a slightly lower aggregate content (lost during service life). Moreover, tear-off MW contains harder and more viscous bituminous binder because of the weathering processes (aging, oxidizing, volatilization, etc.) suffered outdoors during the membranes’ service life [12]. For these reasons, tear-off membrane waste with different characteristics can be available in the market in view of their original composition and as a function of their in-service life conditions.

Generally, there are two different types of polymer-modified bitumens used to produce waterproofing membranes, i.e., Styrene Butadiene Styrene (SBS)- and Atactic PolyPropylene (APP)-modified bitumen. SBS-modified bitumen in waterproofing membranes is used in colder regions due to its high elasticity and elongation, while APP-modified bitumen membranes are preferred in high-temperature climates due to their higher consistency at high temperature with respect to SBS-modified bitumen [7]. In particular, the selected modification technology can be a key topic. Indeed, based on the designed service conditions, high-temperature stability or low-temperature ductility can be alternatively promoted by using APP- or SBS-modified bitumens, respectively. Typically, elastomeric SBS promotes deformation recovery and flexibility at low temperatures, reducing thermal cracking potential while maintaining adequate elasticity and stiffness at high temperatures (good rutting resistance). On the other hand, plastomeric APP generally increases stiffness (providing excellent high-temperature rutting resistance) with limited elasticity, being more prone to low-temperature cracking [20,21].

As a consequence, depending on the recycling dosage, MW type and composition can have a certain influence on the performance of the asphalt mixtures, and its compatibility with the selected virgin bitumen is also considered [22]. For instance, based on material compatibility theory, Lu and Isacsson (1997) reported that recycled SBS-based membranes can be more compatible with SBS-modified virgin binders with respect to APP-modified compounds [23]. Regardless of the binder type, the MW conversion in a valuable construction material is a relatively novel technique. Specific standards and requirements are not yet fully implemented in worldwide technical specifications delivered by agencies, authorities, contractors, etc. In turn, reference international technical standards are still lacking. In this perspective, the current research aims at demonstrating that given adequate selection and treatment procedures of MW (e.g., screening, shredding, blending, watering, etc.), MW-modified asphalt mixtures can perform similar to (or even better than) traditional hot-mix asphalts. This study is a part of a wider in-progress research project aimed at evaluating the possible reuse of MW within asphalt mixtures. In particular, the technical, economical and environment feasibility of recycling MW by-products will be assessed in order to effectively promote the circular economy. Since ready-to-use technical documents for such recycling practices are currently lacking, practical guidelines addressing the maximum allowed recycling rate, the correct recycling process and the needed adaptions for manufacturing, production, laying and compaction of MW-modified asphalt mixtures, as well as the expected performance of the real-scale recycled mixtures, are desirable.

Few studies in the literature exist in this regard. Indeed, Merine et al. assessed the potential of recycled roofing membranes in the laboratory in terms of rheology, stiffness, fatigue and permanent deformation resistance, preparing hot bituminous mixes also containing reclaimed asphalt [18]. With respect to the control mix, those containing the membranes limited the high-temperature strain accumulation, being characterized by stiffer and more elastic bituminous blends. Additionally, some benefits associated with fatigue resistance were also observed. The sustainability and the ecological potential of MW recycling was also demonstrated in a recent study that performed a life cycle assessment on hot asphalt mixtures containing roofing wastes [24]. The authors were able to estimate a more than 10% reduction in global warming impact indicators thanks to a 25% reduction of the virgin bitumen in the mixture as a result of MW inclusion. Moreover, a twenty-times reduction in the fuel consumption for the mixture production and a five-times decrease in the emissions were also calculated.

This paper’s structure consists of four main sections: after the Introduction, the research objectives and approach are detailed; then, the experimental activities (materials, specimens preparation and testing methods) are described; finally, the experimental outcomes are presented and discussed to draw related conclusions.

## 2. Objectives and Research Approach

In this paper, a laboratory research study focusing on a comprehensive characterization of MW-modified asphalt mixtures is presented (end-of-life tear-off membranes). As previously stated, such experimentation is part of a wider research project about MW recycling in bituminous binders and mixtures to produce sustainable pavements by promoting the saving of valuable natural resources and material circularity [25]. Here, the experimental findings related to two MW-modified mixtures are presented.

In particular, MW was dosed at 0.5% by mix weight and 2% by mix weight for the two mixes, respectively; these investigated mixtures were coded as SH− (0.5% by mix weight) and SH+ (2% by mix weight). An amount of 0.5% MW by mix weight corresponds to 10% by binder weight, which is the optimum MW content found in a preliminary study on wet MW-modified bituminous binders [25], while 2% by mix weight was selected as the maximum allowable MW amount without mix design modification. A corresponding reference mix without MW was also tested for comparison purposes (mix coded as REF).

These mixes were subjected to different performance tests, i.e., Indirect Tensile Strength (ITS) tests in both dry and wet conditions, Indirect Tensile Stiffness Modulus (ITSM) and Indirect Tensile Fatigue (ITF) tests and Repeated Load Axial (RLA) tests, to determine their strength, moisture damage resistance, stiffness, fatigue resistance and resistance against permanent deformation, respectively.

## 3. Experimental Program

**Figure 1:**
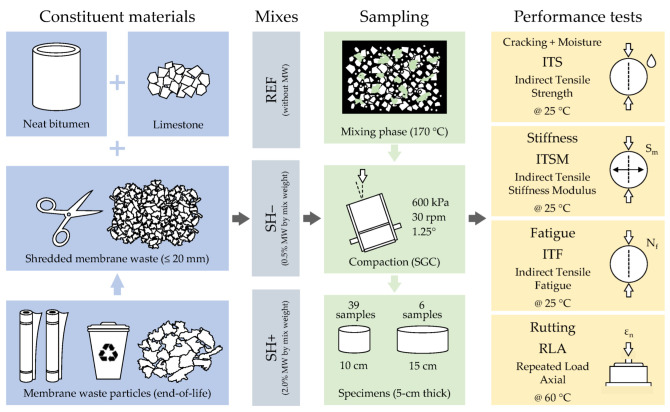
Schematic of the experimental program.

A schematic of the experimental program used in this research study is given in Figure 1 that illustrates materials used, specimen preparation and performed tests. More details are provided in the following corresponding sections.

### 3.1. Material and Specimen Preparation

MW as well as 70/100 penetration neat bitumen were provided by bituminous membrane production companies, while natural limestone aggregates typically used in Italian pavements were taken from a local asphalt production plant (Northern Italy). The basic physical properties of bitumen can be found elsewhere [25], whereas those of aggregates are summarized in Table 1.

**Figure 2:**
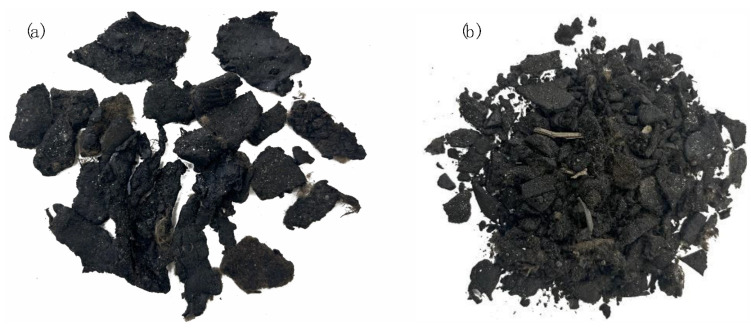
Original MW product (**a**) and shredded MW after cutting (**b**).

In particular, the MW was a proprietary product from end-of-life recycled bituminous roofing felt (Figure 2a). Such bituminous shreds, mainly constituted by bitumen (more than 45% by their total weight) and without foreign matter (less than 1% by volume), were verified to have a softening point lower than 100 °C and a penetration grade higher than 15 × 10^−1^ mm.

Such constituent materials were used to produce in the laboratory 10 mm maximum-aggregate-size dense-graded bituminous mixtures for wearing course, according to Italian technical specifications [26]. In particular, based on a preliminary Marshall mix design, bitumen was dosed at 5.5% by aggregate weight, while aggregates were proportioned combining the four different aggregate stockpiles (named ‘Filler’, ‘0/4’, ‘4/8’ and ‘8/14’) at 5%, 37%, 35% and 23%, respectively (Table 2).

To be used for mixture preparation in this laboratory research, the largest MW shreds were first cut into small pieces manually (20 mm maximum size) to facilitate their homogeneous distribution into the asphalt mixtures prepared by hand in the laboratory (Figure 2b). The cutting operations were executed to make the MW dimensions more compatible with the selected maximum aggregate size (10 mm) while saving operational time, also considering that the majority of the MW particles would likely melt at the asphalt manufacturing temperatures [25]. MW was then stored at ambient temperature prior to being added to the hot (i.e., 170 °C) bitumen and aggregates (dry-addition method). All the materials were then stirred homogenously, placed in the oven for a further 10 min at the same mixing temperature and finally compacted using a Shear Gyratory Compactor (SGC) by imposing a final height of 50 mm (target void content of 5.0%). The available amount of materials allowed the preparation of 6 (2 for each mix) 15 cm diameter cylindrical specimens to be subjected to confined RLA tests and 39 (13 for each mix) 10 cm diameter cylindrical samples for all the other planned tests. In this regard, it is specified that the samples size was due to practical laboratory constraints related to the destructive nature of asphalt testing as well as to experimentation time. Indeed, notwithstanding that this could restrict the general statistical power of the analysis, the results gave useful comparative trends and were consistent with similar experimental protocols presented in a valuable study in the literature that adopted 3 to 5 samples per group [27].

As previously described, a reference mixture (REF) without MW as well as two MW-modified mixes were prepared (SH− and SH+ mixes with 0.5% and 2.0% MW content, respectively); the two MW contents were selected to reproduce both the optimum MW content found in a preliminary study on wet MW-modified bituminous binders [25] and the maximum allowable MW amount without mix design modification.

### 3.2. Testing Methods

#### 3.2.1. Indirect Tensile Strength Test

An Indirect Tensile Strength (ITS) test was performed according to EN 12697-23 on 100 diameter and 50 mm thick cylindrical specimens prepared with the SGC. Four specimens for each mixture were tested at 25 °C by applying a constant vertical loading rate of 51 mm/min (after 4 h conditioning time at the test temperature). The vertical load was continuously monitored through a loading cell while the vertical sample deformation was recoded with a linear variable displacement transducer. The indirect tensile strength was then determined as reported in Equation (1):

ITS (MPa) = 2 · *P*/π · *D* · *t* (1)

where *P* is the applied load at failure (N), while *D* (mm) and *t* (mm) are specimen diameter and thickness, respectively.

#### 3.2.2. Moisture Resistance Evaluation

According to EN 12697-12, moisture resistance was assessed in terms of the Indirect Tensile Strength Ratio (ITSR), that is, the percentage ratio between the ITS of wet-conditioned specimens and the ITS of dry-conditioned specimens (determined according to the previous section). To this aim, a subset of four specimens for each investigated mixture was wet-conditioned by soaking the samples in water at 40 °C for 72 h. Then, specimens were conditioned for at least 4 h at 25 °C prior to testing. Calculation details are given in Equation (2):

ITSR (%) = 100 · *ITS_w_*/*ITS_d_* (2)

where *ITS_w_* (MPa) and *ITS_d_* (MPa) are the indirect tensile strength of the wet-conditioned samples and the dry-conditioned samples, respectively.

#### 3.2.3. Indirect Tensile Stiffness Modulus Test

Indirect Tensile Stiffness Modulus (ITSM) tests were performed at 25 °C according to 12697-26 (Annex C) on 100 mm diameter cylindrical specimens; samples were conditioned at the test temperature for at least 4 h before testing. For each investigated material, such non-destructive tests were performed in strain-controlled mode on all 13 available samples. A load actuator applied the load pulses while the corresponding horizontal deformation was measured through two linear variable displacement transducers mounted opposite one another in a rigid frame clamped to the specimen. A rise time (time for applying the load from zero to load peak) of 124 ms and a target peak horizontal deformation of 5 µm were selected. Then, the stiffness modulus *S_m_* was calculated as the average of 5 load pulses, according to Equation (3):

(3) $$S_{m}\;\left(\mathrm{MPa}\right)=\frac{F\cdot (v+0.27)}{z\cdot h}$$

where *F* (N) is the recorded load, *ν* is the Poisson’s ratio (considered equal to 0.35), *z* (mm) is the amplitude of the resilient horizontal deformation obtained during the load and *h* (mm) is the specimen height.

#### 3.2.4. Indirect Tensile Fatigue Test

Indirect Tensile Fatigue (ITF) tests were performed at 25 °C according to BS DD ABF standard on 100 mm diameter cylindrical specimens after 4 h conditioning at the test temperature. Such destructive tests were performed in stress-controlled mode on 5 available specimens by applying different stress levels ranging from 20 to 120 kPa up to the physical failure of the specimens. The test consisted of the application of selected repeated load pulses in indirect tensile configuration with a haversine pulse characterized by 124 ms risetime. This led to a preliminary indication of fatigue cracking potential, while a more reliable assessment will be carried out in future research properly focused on fatigue. Prior to testing, the indirect tensile stiffness modulus *S_m_* (MPa) was measured in stress-controlled mode in order to calculate the applied initial strain value *ε* according to Equation (4). Fatigue resistance was analyzed in terms of fatigue curves (i.e., number of cycles to failure *N_f_* vs. applied initial strain *ε*).

(4) $$\varepsilon \;\left(\mu \mathrm{strain}\right)=1000\cdot \frac{\tau \cdot (1+v)}{S_{m}}$$

where *τ* (kPa) is the selected stress level for stress-controlled ITSM and fatigue tests.

#### 3.2.5. Repeated Load Axial Test

Resistance to permanent deformation of bituminous mixtures was determined through Repeated Load Axial (RLA) test on 150 mm diameter cylindrical specimens following EN 12697-25 standard. After 4 h conditioning at 60 °C, tests were performed at the same temperature in stress-controlled mode by applying a block-pulse stress (1 s load and 1 s rest) at 300 kPa. A load actuator applied the load pulses while the corresponding vertical deformation was measured through two linear variable displacement transducers mounted axially to the sample geometry. In particular, two specimens for each material were tested by loading them centrically in the axial direction with a 100 mm diameter plate to simulate pavement field confinement conditions. Results were obtained in terms of cumulative strain *ε_n_* vs. applied number of cycles *N*, up to the sample failure (i.e., deformation higher than 10 mm).

## 4. Results and Discussion

The following subsections present the main experimental results and give related discussions about the investigated performance of materials. In this respect, it is worth specifying that all measurements had noticeable repeatability, as shown in Table A1 (Appendix A) with respect to the statistical acceptance of measures described in ASTM C670. Statistical analyses were run through Python (3.13.3 version).

### 4.1. Compactability

**Figure 3:**
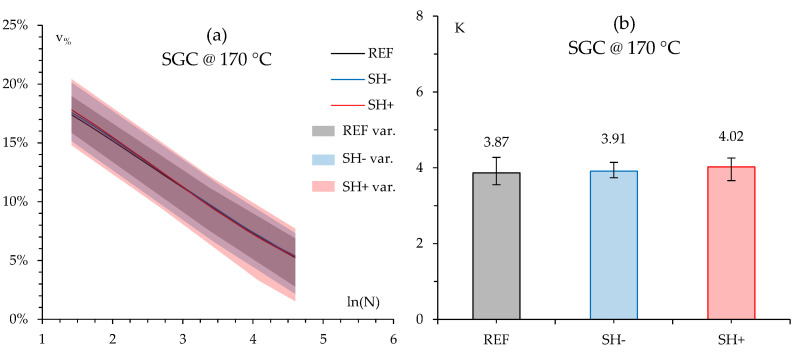
Bituminous mixture compaction curves (**a**) and compactability values K (**b**).

Compaction through the SGC allowed the construction of compaction curves. According to EN 12697-10, such curves were obtained by plotting air voids (*v_%_*) as a function of the natural logarithm of the number of gyrations *N*; their slope identifies the compactability *K*. Compaction curves as well as the corresponding slope (*K* values) for both neat and MW-modified bituminous mixtures are shown in Figure 3a and 3b, respectively. In Figure 3a (as well as in all the following similar graphs), the continuous lines indicate the average results among the test replicates, whereas the corresponding colored areas represent the experimental data variability, i.e., the minimum and maximum measured values among the test replicates. In Figure 3b, the error bars indicate the same experimental data variability.

It is worth noting that the addition of membrane waste (either high-quantity SH+ or low-quantity SH−) in bituminous mixtures does not seem to change the compaction behavior, since similar trends in the compaction curves (i.e., compactability) and final voids of the modified mixtures were observed with respect to the reference neat mixture, as confirmed also by the statistical analysis presented in Table A2 and Table A3 in Appendix A. Based on Shapiro–Wilk tests (Table A2) [28], all data were normally distributed, allowing parametric testing (Shapiro–Wilk *p*-value always above the established 0.05 threshold). Based on this result, an analysis of variance ANOVA at a 95% confidence level (Table A3) was selected, demonstrating that differences between series were not statistically significant. Actually, since the multiple group comparison increases the risk of type error I (i.e., false positive) a Bonferroni correction [29] was also applied to adjust the significance limit (new threshold equal to 0.025 as result of 0.05 divided by the two comparisons performed). Under this assumption, no statistical significance in the observed differences was again found (see further details in Table A3).

Hence, it can be assumed that mixtures with a limited amount of MW (up to 2%) will exhibit acceptable workability during field compaction without arranging specific compaction protocols (i.e., higher temperature and/or rejuvenator/fluxant additions) while allowing desirable recycling of membrane waste.

### 4.2. Tensile Strength

**Figure 4:**
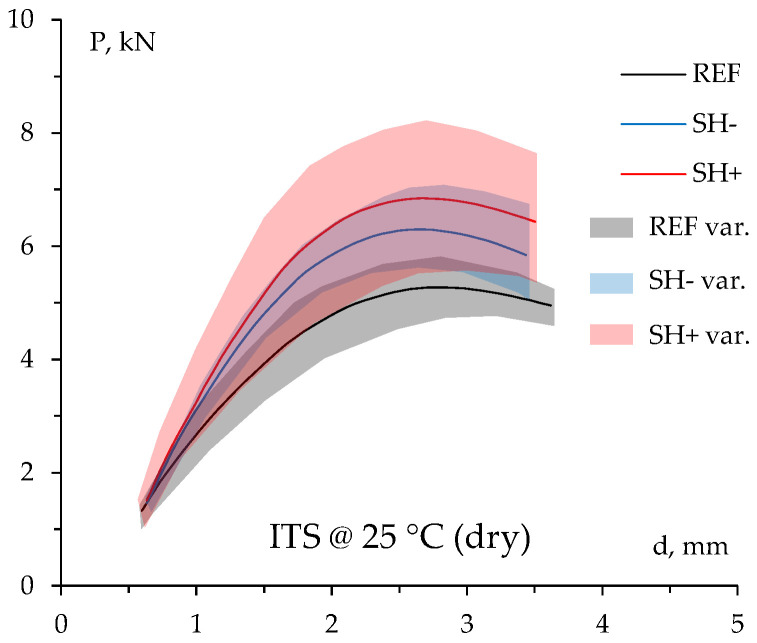
Experimental data of ITS test in dry condition.

**Figure 5:**
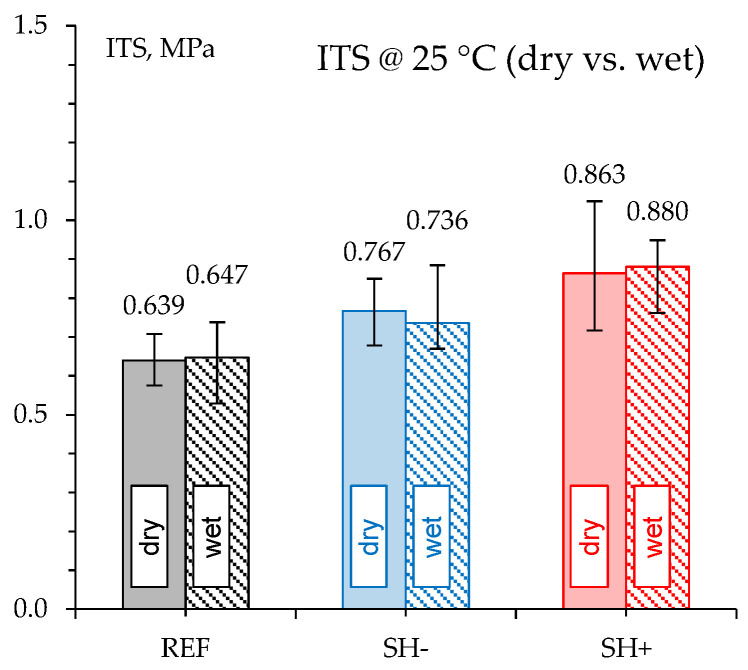
ITS measured in dry and wet conditions.

Figure 4 reports the experimental readings during ITS tests carried out at 25 °C on dry-conditioned samples, while the corresponding indirect tensile strengths are shown in the following Figure 5. As previously explained, the colored areas indicate the minimum and maximum measured experimental data.

As can be observed, the addition of membrane waste led to enhanced indirect tensile strength of the bituminous mixtures (i.e., +20% in the case of 0.5% MW content and +35% in the case of 2% MW content, respectively); a higher ITS was observed in the case of SH+ specimens, likely due to the higher amount of aged (stiffer) bitumen in the mixture (tear-off end-of-life hard MW). Statistics (Table A2 and Table A3 in Appendix A) indicate significant differences in the case of SH+. Such a finding was demonstrated by the ANOVA *p*-value in the case of a corrected Bonferroni threshold (Shapiro–Wilk test again supported the analysis of variance). However, it is pointed out that such increased tensile strength is, in any case, acceptable based on common technical specifications [26], which often establish a maximum limit of about 1.4–1.5 MPa in order to avoid excessive brittleness (likely due to the considerable amount of aged bitumen from RAP in the mixture). In fact, it is well recognized that strength in asphalt mixtures improves load-bearing capacity and reduces permanent deformation (e.g., rutting) even if it can lead to brittleness issues, especially under thermal or fatigue-loading conditions. Such a brittleness increases the risk of cracking, particularly in cold climates or under repeated traffic loading, as it reduces the ability to relax stress, thus promoting brittle fracture [30].

It is also worth noting that the increase in strength detected in this experimental study is noticeably higher than that reported by other researchers in a previous study (i.e., +7%) investigating a similar wearing course mixture prepared with SBS-polymer-modified bitumen and containing 1% by mix weight of APP-based recycled bituminous membrane [31]. Likely, this may be mainly due to the softer base bitumen characterizing the mixes investigated in the present study, which enhanced the stiffening effect of the APP-modified bitumen coming from the selected MW.

### 4.3. Moisture Resistance

Figure 5 depicts the average ITS values and error bars (min. and max. measured values) recorded at 25 °C in both dry and wet conditions (i.e., on specimens placed in a water bath at 40 °C for 72 h prior to testing).

As can be seen, the MW-modified mixes were characterized by higher strength in both dry and wet conditions regardless of the waste content. Moreover, all the tested mixtures revealed similar dry and wet ITS values that led to an ITSR value (i.e., the percentage ratio between ITS in wet condition and ITS in dry condition) close to 100%. It is worth noting that the slight differences observed in terms of average values are only due to the natural variability in fabricated specimens as demonstrated by the reported error bars. Indeed, this experimental finding suggested that the selected water conditioning did not affect the strength of the investigated materials, MW-modified mixes included. This indicates the feasibility of recycling the selected membrane waste into the investigated materials without affecting their durability.

### 4.4. Stiffness

**Figure 6:**
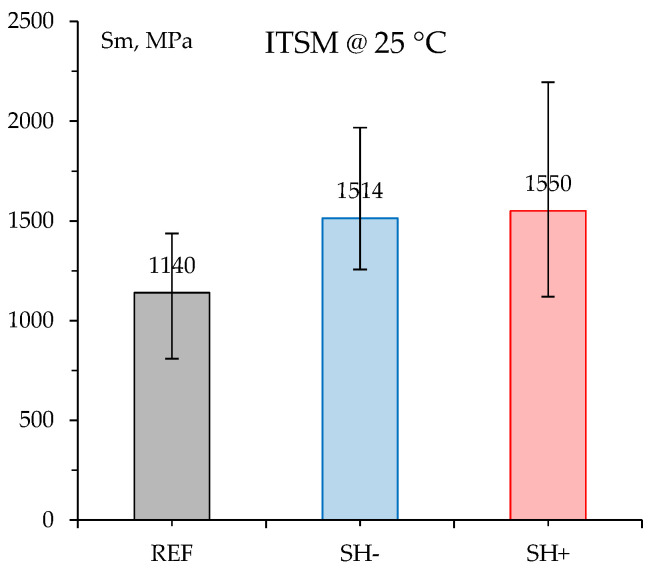
ITSM test results.

The stiffness properties measured in indirect tensile configuration at 25 °C are reported in Figure 6 as average modulus values *Sm* (again, error bars represent the min. and max. experimental data). According to the strength characteristics, the neat specimens showed clear lower stiffnesses than the MW-modified ones, even if high scattering of data was observed. ANOVA results with Bonferroni correction (Table A3 in Appendix A) confirmed the statistical significance of the highlighted differences (Shapiro–Wilk test in Table A2 demonstrated again the reliability of ANOVA). Such a stiffness increase (≈+35%) can be ascribed to the hard aged binder provided to the mixes by the MW shreds. As already discussed, this fact surely helps load distribution within the pavement but could potentially reduce ductility and stress relaxation characteristics (brittle behavior), thus implying that high strength negatively affected the cracking resistance.

Here, it is also worth noting that the stiffness increase experienced by the investigated mixes is very similar to that showed in a previous Italian study where the surface mixture containing 1% recycled bituminous membrane exhibited about a 33% increase in the indirect tensile stiffness modulus with respect to the corresponding reference mixture [32]. Furthermore, the experimental findings illustrated by Merine et al., who carried out four-point-bending cyclic tests on a mixture containing 3% recycled roof membrane and that was prepared with a 70/100 penetration bitumen, also confirm the noticeable stiffness increase obtained through MW addition in the case of a softer base binder in the reference mixture [18].

### 4.5. Fatigue Resistance

**Figure 7:**
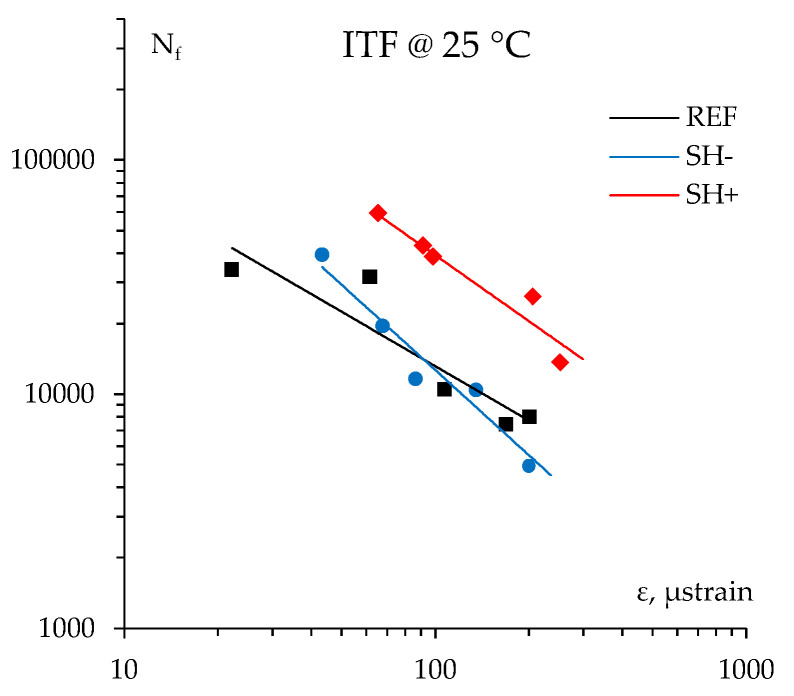
Fatigue lines of the investigated bituminous mixtures.

Figure 7 reports the fatigue lines of the investigated materials obtained by plotting the number of cycles to failure *N_f_* as a function of the initial strain level *ε* from the corresponding stress-controlled indirect tensile fatigue tests. Since only five replicates were available while a consistent fatigue protocol commonly requests at least ten samples, the ITF outcomes should be read as a draft indication of the mixture’s long-term performance and durability. However, despite the few available specimens, good coefficients of determination (*R*^2^) were found for each fatigue line (Table 3), suggesting acceptable reliability.

Again, the presence of MW into asphalt mixtures led to increased performance, especially for the SH+ bituminous mix and at lower strain levels. On the other hand, due to a higher strain sensitivity, the REF mix outperformed SH− and was near SH+ at the higher initial strain levels. This specific behavior of SH+ can surely be ascribed to the presence of a notable amount of harder and more brittle MW bitumen in the asphalt mix. The presence of glass/polyester fibers coming from MW could also provide a positive contribution against cracking in the bituminous phase thanks to a better stress distribution in the mixtures. This likely hindered the expected ductility and crack relief issues, at least for the investigated test conditions. The described outcomes are also supported by the *ε*_6_ values (strain corresponding to 1·10^6^ cycles to failure) presented in Table 3.

Finally, it is worth noting that similar outcomes are illustrated in a previous research study reporting slightly higher fatigue resistance and strain sensitivity (i.e., slope of the fatigue line) for MW-modified surface asphalt mixtures [18].

### 4.6. Permanent Deformation Resistance

**Figure 8:**
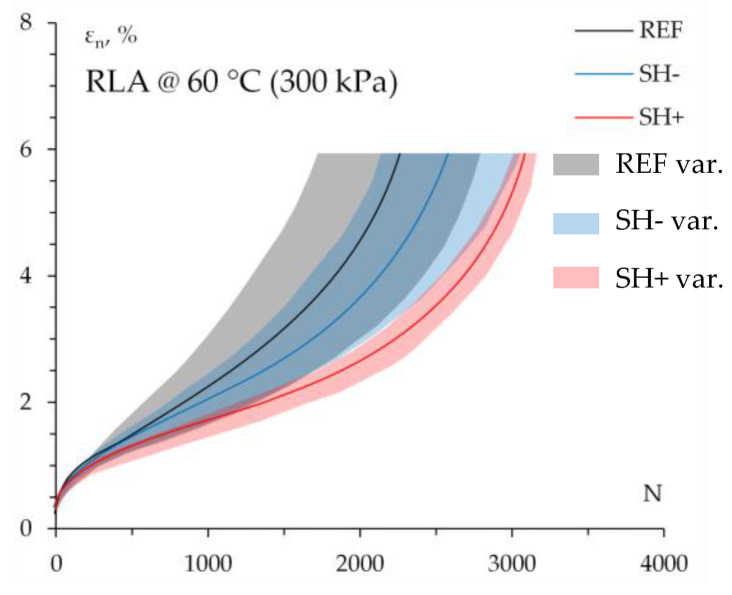
RLA test results.

Creep curves (i.e., permanent axial strain *ε_n_* as a function of number of loading cycles *N*) obtained through RLA tests carried out at 60 °C are reported in Figure 8, where the continuous lines indicate the average values for each mixture, whereas the corresponding colored areas summarize the experimental data variability.

Specimens subjected to 300 kPa block-pulse loading attained the so-called “third stage” of a typical creep curve, i.e., the phase when the slope increases with an increasing number of loading cycles up to failure. This allows us to better differentiate mixture aptitudes against permanent deformation accumulation, revealing that the progressive increase in MW could lead to increasing rutting resistance. The benefit of MW inclusion was numerically demonstrated by the values of creep slope *f_c_* (slope of the linear part of the creep curve) and creep modulus *E*_1800_ (the ratio between the applied stress and the cumulative axial strain after 1800 cycles) that are reported in the following Table 4. Here, moving from REF to SH− and SH+, the lowering of *f_c_* indicated a decreasing susceptibility to deformation as a proportion of the MW contents; at the same time, increasing values of *E*_1800_ demonstrate a noticeable increase in the creep stiffness.

These findings are in accordance with the measured stiffness performance, suggesting that a higher content of stiff aged binder from tear-off MW (see SH+ mix) can help long-term anti-rutting mixture performance, being in line with the existing literature [18].

### 4.7. Discussion

Based on the results presented above, the first promising experimental finding is the unchanged workability of the mix containing up to 2% MW (with respect to the reference material), which suggests no specific strategies for mix design, manufacturing, laying or compaction practices. Analogous considerations can be drawn for the measured performance, since the selected APP membrane waste did not cause significant drawbacks (adequate mechanical performance with comparable or even improved behavior with respect to the REF mix were found in terms of strength, stiffness, moisture, rutting and fatigue resistances). The highlighted results should be definitely reassessed for higher waste content in the mixture, but at the current research stage, encouraging indications towards sustainability are shown.

In this regard, it is worth noting that the current recycling rate (2% by mix weight) could promote a similar reduction of virgin bitumen added, which is reflected in proportional economical savings (up to 10%) towards the circularity of the materials [31]. Similarly, the literature qualitatively reports that incorporating MW into asphalt mixtures can lead to clear reductions in environmental impacts (energy consumption, emissions) associated with bitumen savings [31,33], even if some preliminary steps could be requested (screening, sorting, crushing, cutting, etc.) during real-scale production. In this sense, some studies quantified the costs, energy consumption and gas emissions connected to these preliminary operations, demonstrating that they can have a substantial role in the overall evaluation of recycling sustainability [33,34]. Thus, further analysis is suggested to verify if such impacts could be counterbalanced by the reported benefits due to savings associated with MW addition.

The fundamental aspects will be addressed by the upcoming research continuation, with our further objectives as follows: (i) assessing the feasibility of a further increase in the recycling rate of MW, properly considering real-scale production to achieve feasible engineering practices; (ii) defining appropriate guidelines for all the involved processes in MW recycling; (iii) evaluating the MW compatibility with further materials typically included in asphalt mixtures (such as additives, reclaimed asphalt, etc.); (iv) describing the final in-service short- and long-term performance of MW-modified asphalts (e.g., effects of aging, UV and rainfall, of glass/polyester fibers, etc.). The research approach will properly consider MW characteristics (chemical composition, asphalt content, polymer type distribution, fiber orientation, etc.) as well as the MW–asphalt compounds (microstructure and morphology, bituminous fractions, chemical interactions, interfacial bonding mechanisms, etc.), which should clarify the corresponding macroscopic outcomes.

## 5. Conclusions

This paper presents a part of a wider research project aimed at investigating the feasibility of recycling end-of-life waterproofing bituminous membrane waste in paving bituminous mixtures. In this laboratory step, different amounts of membrane waste were added to asphalt mixtures using the dry method (i.e., 0.5% and 2% by mix weight). A corresponding neat bituminous mixture was also investigated for comparison purposes. Based on laboratory test results, that the following conclusions can be drawn:

- The addition of membrane waste (either 0.5% or 2% by weight of mix) did not affect the workability and the moisture resistance of the investigated bituminous mixtures;
- MW-modified bituminous mixtures were characterized by higher strength, stiffness and permanent deformation resistance with respect to the corresponding reference material; overall, the higher the MW content, the higher the performance increase;
- MW shreds led to a positive effect also regarding the fatigue cracking resistance at lower strain levels; however, MW-modified materials were characterized by a higher strain sensitivity, likely due to the presence of stiff brittle binder.

Hence, promising results were achieved in this preliminary research study about the feasibility of recycling end-of-life bituminous membranes in asphalt mixtures for paving applications using the dry-mixing method. Indeed, the presence of membrane waste does not affect or even increase the investigated asphalt mixtures’ performance, while it allowed a significant recycling rate (2%), thus sensibly promoting the circular economy in transport infrastructures.

As discussed, more research is in any case needed in both the laboratory and the field, which is also due to the noticeable variability in the available market products. First, such preliminary findings need to be confirmed by using different raw materials and mixture types. Then, further specific research should address cracking resistance and ductility issues and also monitor the performance under long-term conditions. Moreover, plant and field mix preparation and application should be promoted in order to verify the actual technical and economic feasibility of producing and applying such a modified mixture (e.g., timing, equipment, procedures, etc.) to try to bridge the gap in knowledge about scalability at the industrial scale. In this sense, it is worth mentioning that a preliminary promising plant-scale production has been carried out by adding MW to a conventional asphalt mixture whose performance is currently under investigation in the laboratory. All such concerns will be implemented to further study the technical, economical and environmental feasibility of MW recycling, establishing practical guidelines to maximize effectively the allowed recycling rate.

## Figures and Tables

**Table 1:** Basic properties of aggregates.

Property	Standard	8/14	4/8	0/4
Shape Index, %	EN 933-4	5.94	8.23	-
Flakiness Index, %	EN 933-3	10.52	7.19	-
Sand Equivalent, %	EN 933-8	-	-	81
Particle density, g/cm^3^	EN 1097-6	2.73	2.54	2.55
Los Angeles, %	EN 1097-2	21	-	-
Water absorption, %	EN 1097-6	0.59	0.79	1.30

**Table 2:** Aggregate and mixture gradation.

Sieve Size, mm	Stockpiles, % Passing				Mix,	Specs [26],
	Filler	0/4	4/8	8/14	% Passing	% Passing
12.5	100	100	100	100	100	100–100
10	100	100	100	79	95	90–100
8	100	100	96	31	83	70–90
6.3	100	100	71	6	68	-
4	100	95	16	1	46	40–55
2	100	62	4	1	29	25–38
1	100	43	1	1	21	-
0.5	100	30	0	1	17	14–20
0.25	100	20	0	1	13	10–15
0.125	99	13	0	0	10	-
0.063	94	11	0	0	9	6–10

**Table 3:** Main characteristics of the fatigue lines.

Mixture	*K*_1_	*K*_2_	*R* ^2^	*ε*_6_, µstrain
REF	462,219	0.773	0.72	0.887
SH−	3,321,217	1.209	0.96	3.793
SH+	2,923,936	0.936	0.81	5.278

Fatigue law: *N_f_* = *K*_1_·*ε*^−*K*2^ *ε*_6_: strain at 1·10^6^ cycles to failure.

**Table 4:** Main characteristics of the creep curves.

Mixture	*N_f_*, Cycles	*f_c_*, µstrain/Cycle	*E*_1800_, MPa
REF	2350	17.19	8.60
SH−	2600	13.49	9.79
SH+	3100	8.64	14.02

## Data Availability

The original contributions presented in this study are included in the article. Further inquiries can be directed to the corresponding author.

## Appendix A

**Table A1:** Measurement repeatability.

Property	Parameter	Unit	Series	*N*	*σ*	2*ds*	*var*	Reproducible?
Compactability	*K*	-	REF	13	0.189	0.850	0.718	Yes
			SH−		0.119	0.534	0.399	Yes
			SH+		0.162	0.728	0.600	Yes
Tensilestrength	*ITS*	MPa	REF (dry)	4	0.073	0.263	0.132	Yes
			REF (wet)		0.098	0.354	0.209	Yes
			SH− (dry)	4	0.082	0.297	0.173	Yes
			SH− (wet)		0.100	0.361	0.216	Yes
			SH+ (dry)	4	0.158	0.570	0.333	Yes
			SH+ (wet)		0.082	0.297	0.187	Yes
Stiffness	*ITSM*	MPa	REF	13	170	765	627	Yes
			SH−		224	1007	712	Yes
			SH+		327	1470	1074	Yes

*N*: number of repetitions, *σ*: standard deviation, 2*ds*: max. acceptance range, *var*: max. effective range.

**Table A2:** Shapiro–Wilk checks for validity of parametric testing.

Property	Parameter	Unit	Series	*N*	W-Statistics	*p*-Value	Verified? *
Compactability	*K*	-	REF	13	0.9546	0.6689	Yes
			SH−	13	0.9407	0.4663	Yes
			SH+	13	0.9459	0.5382	Yes
Tensilestrength	*ITS*	MPa	REF	4	0.7654	0.0533	Yes
			SH−	4	0.9068	0.4658	Yes
			SH+	4	0.9025	0.4438	Yes
			REF	4	0.9095	0.4800	Yes
			SH−	4	0.7582	0.0518	Yes
			SH+	4	0.8662	0.2831	Yes
Stiffness	*ITSM*	MPa	REF	13	0.9898	0.9996	Yes
			SH−	13	0.8860	0.0859	Yes
			SH+	13	0.9760	0.9540	Yes

*N*: sample size. * Normality assumption met (validity of parametric testing).

**Table A3:** Analysis of variance (ANOVA) of experimental findings.

Property	Parameter	Unit	Series	DF	SS	MS	*F*	*p*-Value	Significant? *
Compactability	*K*	-	REF vs. SH−	25	0.000038	0.000003	2.0498	0.1651	No
			REF vs. SH+	25	0.000077	0.000023	4.2603	0.0510	No
Tensilestrength	*ITS*	MPa	REF vs. SH−	7	0.069421	0.033025	5.4443	0.0584	No
			REF vs. SH+	7	0.191811	0.100543	6.6098	0.0423	No
			REF vs. SH−	7	0.074752	0.015584	1.5803	0.2554	No
			REF vs. SH+	7	0.157869	0.108504	13.1879	0.0109	Yes
Stiffness	*ITSM*	MPa	REF vs. SH−	25	1522617	746648	23.0931	0.0001	Yes
			REF vs. SH+	25	2913961	1074278	14.0147	0.0010	Yes

DF: degree of freedom, SS: sum of square, MS: mean square. * Significance check considers the adjusted acceptance threshold through Bonferroni correction (α = 0.025).
